# Supplementary figures and images for: Structural basis for hemoglobin scavenging by CD163 reveals mechanism of ligand promiscuity
Source: PLoS Biol. 2026 May 14;24(5):e3003788. doi: 10.1371/journal.pbio.3003788 (PMC13175321; doi:10.1371/journal.pbio.3003788)

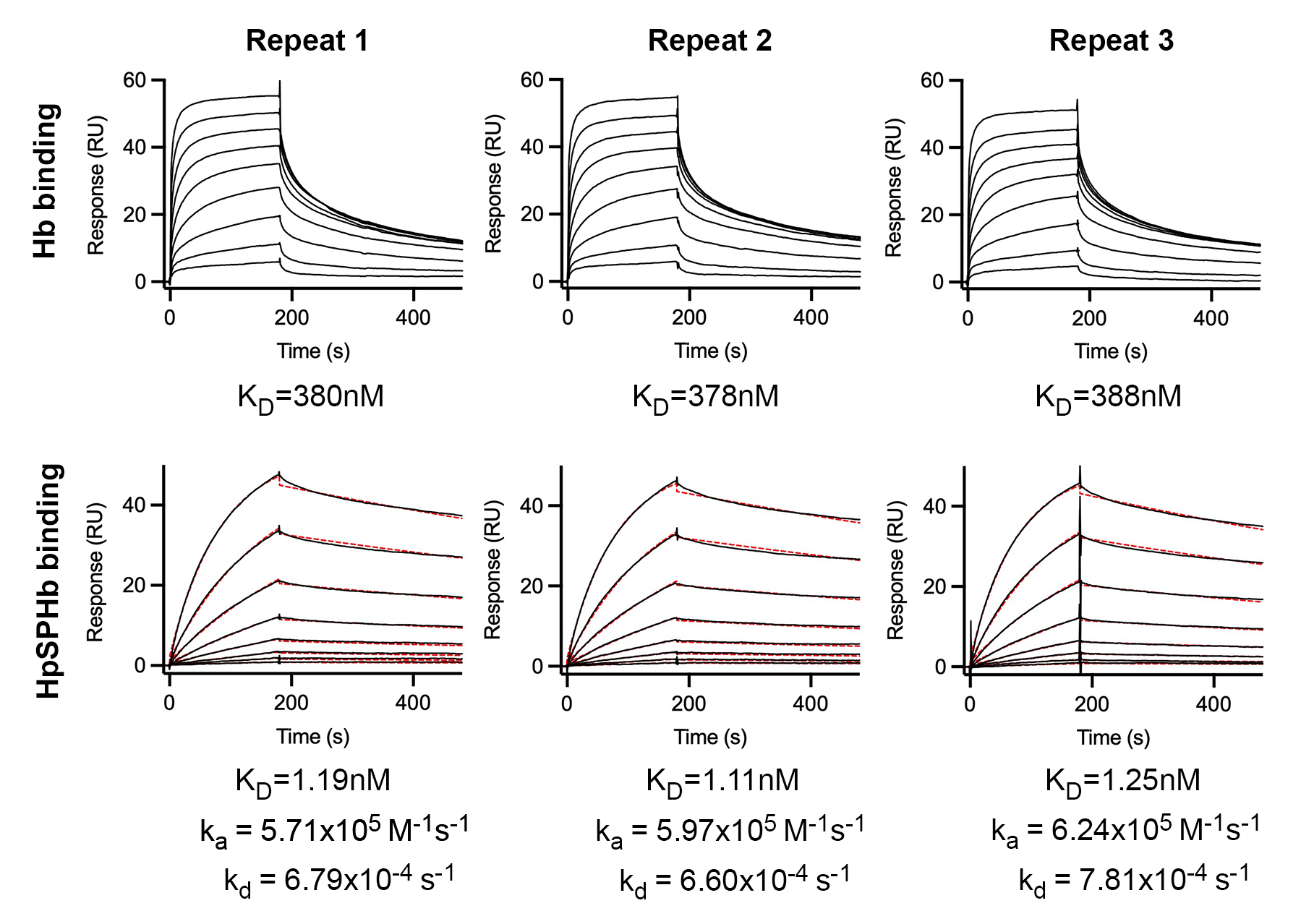

Supplement: S1 Fig — Surface plasmon resonance traces for binding of Hb (top) and HpSPHb (bottom) to immobilized CD163, each shown in triplicate. In the case of Hb, a 2-fold dilution series was used from a top concentration of 16 μM and data was fitted using equilibrium fitting. In the case of HpSPHb, a 2-fold dilution series was used from a top concentration of 20 nM. Data was fitted to a 1-to-1 binding model (red dashed lines), and the binding parameters are shown below the curves. The underlying data can be found in S1 Data. (TIF) [file pbio.3003788.s001.tif]

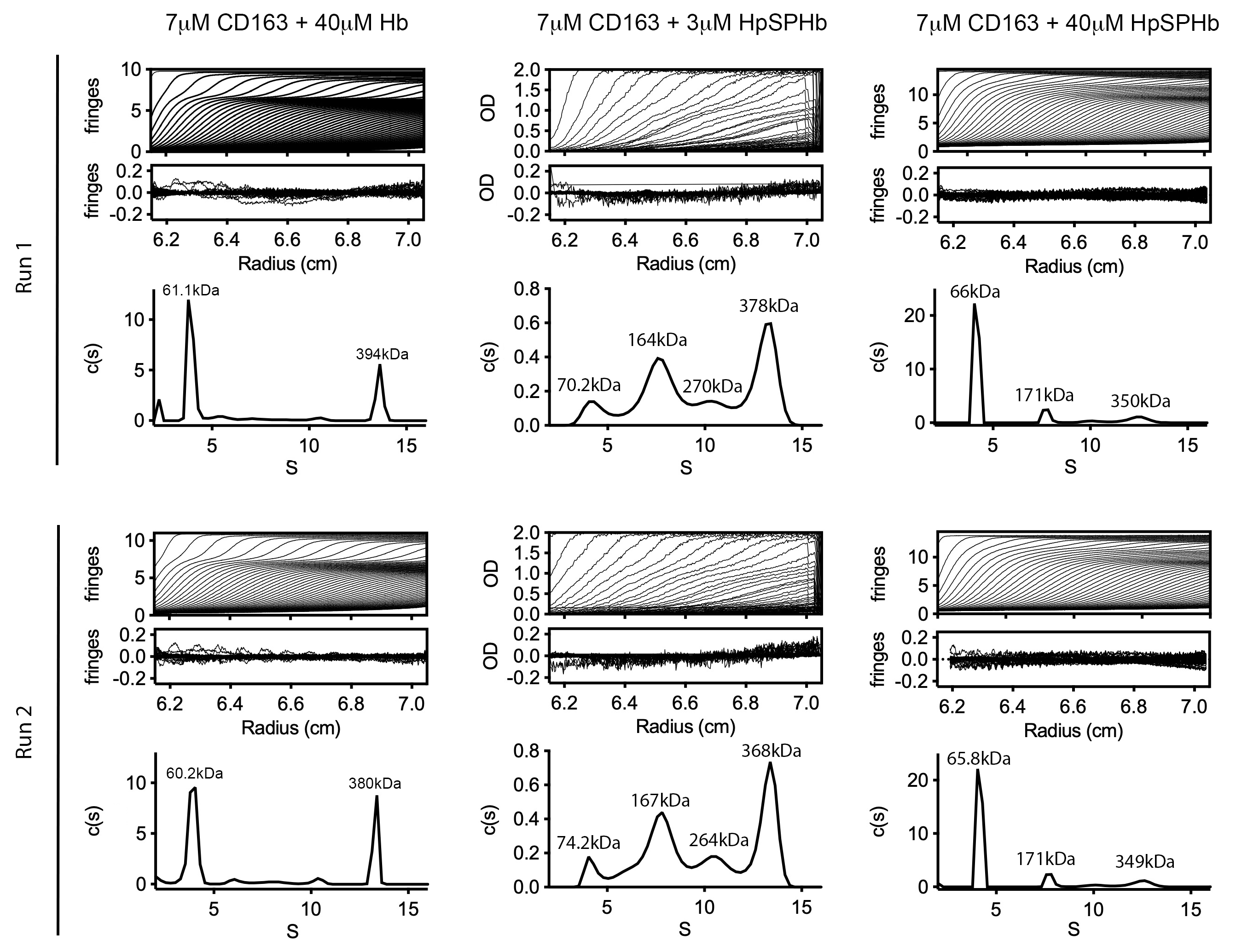

Supplement: S4 Fig — Analytical ultracentrifugation data for 7 μM CD163 in complex with 40 μM Hb (left), 3 μM HpSPHb (center), or 40 μM HpSPHb (right). In each case, two independent runs were conducted (top and bottom). Each set of three panels shows raw data (top), residual after fitting (middle), and fitted distribution data (bottom). Approximate molecular weights are shown above their corresponding peaks. The underlying data can be found in S1 Data. (TIF) [file pbio.3003788.s004.tif]
