# Supplementary material for: Structural basis for hemoglobin scavenging by CD163 reveals mechanism of ligand promiscuity
Source: PLoS Biol. 2026 May 14;24(5):e3003788. doi: 10.1371/journal.pbio.3003788 (PMC13175321; doi:10.1371/journal.pbio.3003788)
Supplement: S1 Table — (DOCX) [file pbio.3003788.s005.docx]

***S1 Table Cryo-EM data collection, refinement and validation statistics***

|  | Map 1 (EMD-56136) | Local refinement map 1 (EMD-56137) | Map 2  (EMD-56138) | | Composite map  (EMD-56135) | |  |
| --- | --- | --- | --- | --- | --- | --- | --- |
| **Data collection and processing** | | |  |  | |  |  |
| Microscope | Titan Krios  Gatan K3 with BioQuantum Imaging Filter  58,149  300  38.30  -0.6 to -1.8  0.832  C1  11,892,554 | | |  | |  |  |
| Detector |  |  |  |  | |  |  |
| Magnification |  |  |  |  | |  |  |
| Voltage (kV) |  |  |  |  | |  |  |
| Electron exposure (e-/Å^2^) |  |  |  |  | |  |  |
| Defocus range (μm) |  |  |  |  | |  |  |
| Pixel size (Å) |  |  |  |  | |  |  |
| Symmetry imposed |  |  |  |  | |  |  |
| Initial particle images (no.) |  |  |  |  | |  |  |
| Final particle images (no.) | 1,033,649 | 1,033,649 | 477,310 |  | |  |  |
| Map resolution (Å) | 2.79 | 3.10 | 3.08 |  | |  |  |
| FSC threshold 0.143 | |  |  |  | |  |  |
| Map resolution range (Å) | 2.32 - 44.9 | 2.61 – 45.7 | 1.79 – 47.7 |  | |  |  |
| FSC threshold 0.5 | |  |  |  | |  |  |
| Map combination method | |  |  | ChimeraX | |  |  |
|  | |  |  |  | |  |  |
|  |  |  |  |  | |  |  |
| **Refinement** |  |  |  |  | |  |  |
| Initial model used |  | |  | AlphaFold2, 9HEK, 1HHO | |  |  |
| Model resolution (Å) |  |  |  |  | |  |  |
| FSC threshold 0.5 |  |  |  | 3.5 | |  |  |
| Model composition |  |  |  |  | |  |  |
| Protein residues |  |  |  |  | |  |  |
| Ligands |  |  |  | 3110 | |  |  |
| R.m.s deviations | |  |  |  | |  | |
| Bond lengths (Å) |  |  |  | 0.004 | |  |  |
| Bond angles (°) |  |  |  | 0.708 | |  |  |
| Validation |  |  |  |  | |  |  |
| MolProbity score |  |  |  | 2.3 | |  |  |
| Clashscore |  |  |  | 11.23 | |  |  |
| Rotamer outliers (%) |  |  |  | 2.78 | |  |  |
| Ramachandran plot | |  |  |  | |  | |
| Favoured (%) |  |  |  | 93.5 | |  |  |
| Allowed (%) |  |  |  | 6.5 | |  |  |
| Disallowed (%) |  |  |  | 0 | |  |  |
| Model vs. data fit | |  |  |  | |  | |
| CC (mask) |  |  |  | 0.66 | |  |  |
| CC (box) |  |  |  | 0.70 | |  |  |
