# Supplementary material for: Structural basis for hemoglobin scavenging by CD163 reveals mechanism of ligand promiscuity
Source: PLoS Biol. 2026 May 14;24(5):e3003788. doi: 10.1371/journal.pbio.3003788 (PMC13175321; doi:10.1371/journal.pbio.3003788)
Supplement: S2 Table — (DOCX) [file pbio.3003788.s006.docx]

|  |  |  | **Hb-bound CD163 trimer** | | **HpHb-bound CD163 trimer** | |
| --- | --- | --- | --- | --- | --- | --- |
| CD163 residue | Residue part | Interaction | Ligand residue | Residue part | Ligand residue | Residue part |
| D185(A) | OD1/OD2 | Ca^2+^ coordination |  |  | Hp(K262) | NZ |
| D186(A) | OD1/OD2 | Ca^2+^ coordination |  |  | Hp(K262) | NZ |
| E216(A) | OE1/OE2 | Salt bridge |  |  | Hp(R252) | NH1/NH2 |
| D224(A) | OD1/OD2 | Ca^2+^ coordination |  |  | Hp(K262) | NZ |
| D225(A) | OD1/OD2 | Ca^2+^ coordination |  |  | Hp(K262) | NZ |
| N247(A) | OD1 | Ca^2+^ coordination |  |  | Hp(K262) | NZ |
| N247(A) | ND2 | Hydrogen bond |  |  | Hp(E261) | OE1 |
| D249(A) | OD2 | Salt bridge |  |  | Hp(R252) | NE |
| D249(A) | OD1 | Hydrogen bond |  |  | Hp(S257) | OG |
| D249(A) | OD2 | Hydrogen bond |  |  | Hp(T258) | N/OG1 |
| E252(A) | OD1/OD2 | Ca^2+^ coordination |  |  | Hp(K262) | NZ |
| D292(A) | OD1/OD2 | Ca^2+^ coordination | Upper Hbβ(K95) | NZ | Hbα(K12) | NZ |
| D293(A) | OD1/OD2 | Ca^2+^ coordination | Upper Hbβ(K95) | NZ | Hbα(K12) | NZ |
| H348(A) | NE2 | Hydrogen bond | Heme of upper Hbβ | O1A |  |  |
| Y354(A) | Side chain | Hydrophobic, H-bond |  |  | Hbα(K8) | Side chain |
| Y354(A) | O | Hydrogen bond | Upper Hbβ(K95) | NZ |  |  |
| Y354(A) | OH | Hydrogen bond | Upper Hbβ(K66)/Heme of upper Hbβ | NZ/O1D | Hbα(D75) | OD2 |
| E359(A) | OE1/OE2 | Ca^2+^ coordination | Upper Hbβ(K95) | NZ | Hbα(K12) | NZ |
| T461(A) | OG1 | Hydrogen bond |  |  | Hbα(K17) | NZ |
| D463(A) | OD1/OD2 | Salt bridge |  |  | Hbα(K17) | NZ |
| H464(A) | NE2 | Hydrogen bond | Lower Hbβ(V1)  Lower Hbβ(H2) | N  ND1 |  |  |
| Y465(A) | Side chain | π-π stacking | Lower Hbβ(H2) | Side chain |  |  |
| E216(B) | O | Hydrogen bond | Lower Hbα(K90) | NZ |  |  |
| D249(B) | OD1/OD2 | Salt bridge | Lower Hbα(H45) | ND1/NE2 |  |  |
| H250(B) | ND1/NE2 | Hydrogen bond | Lower Hbα(H45) | ND1/NE2 |  |  |
| D293(B) | OD2 | Salt bridge | Lower Hbα(K60) | NZ | Hbβ(K67) | NZ |
| D293(B) | OD1/OD2 | Hydrogen bond |  |  | Heme of Hbβ | O1A/O2A |
| H353(B) | O | Hydrogen bond |  |  | Hbβ(K96) | NZ |
| Y354(B) | Side chain | Hydrophobic |  |  | Hbβ(L97) | Side chain |
| Y354(B) | O | Hydrogen bond | Lower Hbα(K60) | NZ |  |  |
| D463(B) | OD1/OD2 | Salt bridge |  |  | Hbβ(H78) | NE2 |
| H464(B) | ND1 | Hydrogen bond | Lower Hbα(H20) | ND1/NE2 | Hbβ(A77) | O |
| Y465(B) | OH | Hydrogen bond |  |  | Hbβ(D74) | OD1 |
| D293(C) | OD1/OD2 | Salt bridge | Upper Hbα(K90) | NZ | Hbα(K91) | NZ |
| D331(C) | OD2 | Salt bridge | Upper Hbα(K90) | NZ |  |  |
| K346(C) | NZ | Hydrogen bond |  |  | Hp(N148) | OD1 |
| H347(C) | O | Hydrogen bond |  |  | Hp(S150) | N |
| K352(C) | NZ | Hydrogen bond | Heme of upper Hbα | O1A |  |  |
| Y354(C) | OH | Hydrogen bond | Heme of upper Hbα | O1A | Hbα(H90 and D86) | ND1 or OD1/OD2 |
| Y354(C) | Side chain | Hydrophobic | Upper Hbα(L86) | Side chain |  |  |
| N356(C) | OD1/ND2 | Hydrogen bond | Upper Hbα(H45) | NE2 |  |  |
| E359(C) | OE2 | Salt bridge |  |  | Hbα(K91) | NZ |
| R400(C) | NH1/NH2 | Hydrogen bond |  |  | Hbα(P45) | O |
| R400(C) | NH1 | Salt bridge | Lower Hbβ(D47) | OD2 |  |  |
| D463(C) | OD1/OD2 | Hydrogen bond | Lower Hbβ(S44) | O |  |  |
| D463(C) | OD1/OD2 | Salt bridge | Lower Hbβ(K59) | NZ | Hbα(H46) | ND1/NE2 |
| Y465(C) | OH | Hydrogen bond | Lower Hbβ(D47/N57) | OD1/ND2 | Hbα(H46) | ND1/NE2 |
