# Supplementary material for: Structural basis for hemoglobin scavenging by CD163 reveals mechanism of ligand promiscuity
Source: PLoS Biol. 2026 May 14;24(5):e3003788. doi: 10.1371/journal.pbio.3003788 (PMC13175321; doi:10.1371/journal.pbio.3003788)
Supplement: Attachment — Submitted filename: response to reviewers.pdf [file pbio.3003788.s010.pdf]

Reviewer #1: The scavenger receptor CD163 clears free hemoglobin (Hb) during intravascular hemolysis thereby preventing oxidative damage. Typically, this process is effectively facilitated by the haptoglobin (Hp), which mediates a high-affinity complex formation between CD163 and Hb (termed CD163-HpHb). However, the tolerance observed in patients with genetic absence of Hp, or its conditional depletion during infection or sickle cell anaemia, suggests the existence of an Hp-independent, CD163-depedent clearance mechanism for Hb overload. In this study, the authors provide critical structural insights into this alternative CD163-Hb interaction. They demonstrate that the Hb tetramer engages exclusively with a trimeric form of CD163, in a pattern broadly similar to that of the HpHb ligand, despite a ~300-fold weaker affinity. A subtle difference lies in the local conformational rearrangement of ectodomains SRCR1/2, which may confer the flexibility needed for CD163 to recognize a variety of ligand configurations.

Overall, the manuscript is well-structured, methodologically rigorous, and addresses a biologically important question. The conclusions are supported by the data, and the comparative analysis with previous structural work strengthens the novelty of the findings. This study is suitable for publication in PLOS Biology as a Short Report, pending minor revisions to address the following points:

We thank the reviewer for their positive comments about the manuscript.

A major finding of this study is the ligand-dependent selectivity in the oligomerization state of CD163. The cryo-EM and analytical ultracentrifugation data convincingly show that the Hb tetramer interacts only with CD163 trimer. Would it be feasible to further test the in vitro binding affinity and cellular uptake efficiency of monomeric or dimeric CD163 with Hb tetramer?

We thank the reviewer for noting that our biophysical data convincingly shows that we observed only trimers of CD163 to bind to haemoglobin at the concentrations tested *in vitro*.

The analysis of uptake of different forms of Hb and Hp complexes, including Hb tetramer, was already shown in our previous manuscript on the CD163 structure (Zhou et al (2025) Nature Comms 16 6623), in which we presented uptake data for a monomeric mutant of CD163. While Hb could compete for the uptake of Hp(2-2)Hb into HEK293 cells transfected with wild-type CD163 (Figure 3f), it could not complete when the monomeric mutant was used (Figure 5e). We attributed this to the lower affinity of CD163 for Hb than for HpHb. This means that one arm is enough for functionally effective binding to HpHb, but not to Hb. We have added a sentence to lines 127-130 to point readers to this previously published data and how its conclusion relates to the current discoveries.

Sadly, CD163 adopts a range of multimeric states and we have not found a way to generate a constitutive dimer of CD163 through mutation. We did consider options, such as generating a tandem of two subunits in one polypeptide, but the N- and C-termini lie at opposite ends of the molecule, making this impossible. It has therefore not been technically possible to produce a dimeric CD163 to test ligand binding or uptake.

Nevertheless, our finding that only trimeric ligand-bound complexes are observed in the presence of Hb is consistent with our model, including previous data from the monomeric mutant, which suggests that lower affinity ligands require a trimeric CD163, while monomeric and dimeric CD163 can allow uptake of higher affinity ligands.

In Figure 3D, the label "Hb-bound dimer" should be corrected to "Hb-bound trimer". Furthermore, while the authors compared unliganded CD163 dimer with Hb-bound CD163 trimer, it would be informative to also include a comparison with the HpHb-bound CD163 dimer solved previously by the authors.

We thank the reviewer for spotting this labelling error and we have corrected it.

However, we prefer not to add the structure of the HbHp-bound dimer into Figure 3 as our view is that this will complicate the figure without adding to our conclusions. We could include the dimer structure in panel A but our view is that a comparison of the trimers is clearer. Addition of the HpHb-bound dimer in panel D would not fit with the aim of this panel, which is to compare different arm-arm interactions, as no such interactions are seen in the HpHb-bound dimer.

In Figure S1, it is suggested that the binding parameters between Hb and CD163 be shown for each individual experiment, as is currently done for HpSPHb/CD163.

We have now added the K_D values into this figure. As mentioned in the legend, the Hb data was fitted using equilibrium fitting and so association and dissociation rates were not derived.

Reviewer #2: In this manuscript, the authors investigate how the scavenger receptor CD163 binds and detoxifies free haemoglobin (Hb) during haptoglobin depletion. Using surface plasmon resonance and cellular uptake assays, they confirm CD163 binds and internalizes tetrameric Hb with submicromolar affinity (Kd = 382 nM), compared to its tighter binding to the HpSPHb complex (Kd = 1.18 nM). Cryo-EM analysis reveals a distinct 3:1 binding stoichiometry, demonstrating that a complete trimeric CD163 assembly is required to bind a single Hb tetramer, a finding further validated by analytical ultracentrifugation. By elucidating the structural basis for CD163's "ligand promiscuity," this study explains how the receptor adapts to clear toxic Hb and prevent oxidative damage, making it a compelling contribution for PLOS Biology readers.

We thank the reviewer for their positive comments about our discovery.

While the structural insights presented are valuable, the manuscript currently lacks essential cryo-EM validation metrics. The omission of Fourier shell correlation (FSC) curves, angular distributions, and local-resolution maps precludes a thorough evaluation of the map and model quality. Additionally, several structural figures lack sufficient detail and clarity for readers to properly interpret the authors' claims. Because these are fundamental requirements for any structural biology publication, I recommend a major revision. The authors must address the following points before the manuscript can be considered suitable for publication.

We are sorry to hear that the reviewer was unable to access the data required to assess the quality of the maps. We had included full PDB and EMDB validation reports with the submission, which are considered by the community to be what is required to assess the quality of structural studies. We now include an additional supplementary figure to provide the extra information requested. We have also requested release of coordinates and maps.

Major Revisions

• The manuscript currently lacks essential cryo-EM validation data, preventing a thorough assessment of map and model quality. The authors must provide Fourier shell correlation (FSC) curves, angular distribution plots, and local-resolution maps for Map 1, the local refinement of Map 1, and Map 2. These metrics should be included as supplementary figures.

• In Lines 102-103, the authors state, "The CD163 base, CD163 arms of subunits A and B and the Hb tetramer were well resolved, while arm of subunit C was less well resolved." Without the accompanying local-resolution maps requested above, the terms "well resolved" and "less well resolved" are subjective and difficult to interpret. Providing local-resolution maps colored directly onto the surface representation using tools like Chimera or ChimeraX will clarify these statements.

We have added an additional supplementary figure (Figure S3) to provide this information.

• In Lines 106-107, the authors claim that domains SRCR5-9 form a triangular base through calcium-mediated interactions, similar to the HpHb-bound structure. However, no structural illustrations are provided to support this. The authors should include a figure panel either in the main figures or supplementary figures demonstrating these specific calcium-mediated interactions within the CD163-Hb complex.

It is not our view that the manuscript would benefit from a figure showing molecular models and highlighting these calcium-mediated interactions. The calcium ions at these interfaces are not a discovery of this study but were proposed in three previous studies of HpHb-bound CD163. In fact, it has not formally been demonstrated that these spheres of density are calcium as, unlike in crystallography, it is not possible, for example through anomalous scattering, to formally demonstrate this. However, the reviewers of these three papers were happy to accept that these ions are calcium, based on the requirement for calcium to allow trimer formation and the chemistry of coordination. We have made this point in this manuscript too, by being more circumspect in line 152 where we attribute a sphere of density in the ligand binding site to calcium.

The aim of the paragraph in lines 107-112 is to compare the overall base architecture and arrangement in HpSPHb- and Hb-bound CD163 and our view is that the current representation in Figure 2B shows this most clearly, without more complex and detailed figures of the interface and putative calcium ions, which do not make this point. We therefore prefer to the leave this figure as originally presented.

• Figure 3B is intended to demonstrate the interactions between the CD163 arms and the Hb subunits, but it falls short. Crucially, the authors rely solely on EM maps to illustrate these interfaces. Because map contours can be adjusted, this visual representation can be misleading when assessing whether two entities are genuinely interacting. Furthermore, the specific calcium-ion-mediated interaction referenced in Lines 145-146 is not visible. The authors should update Figure 3B to display the fitted atomic models within the transparent EM density, explicitly highlighting the key interacting residues and calcium ions. Example of such figures can be found in two papers reporting cryo-EM structures of CD163-HpHb complex: https://doi.org/10.1038/s41467-024-55171-4 and https://doi.org/10.1371/journal.pbio.3003264

We strongly disagree with the reviewer that our figures 'fall short'. Different authors prefer to present their structural biology findings using different types of representation which they think best allows communication of their conclusions to the broad readership. Our view is that our current figures do this effectively and that the format proposed by the reviewer would be overly complex and less clear. The reviewer highlights Figure 3B. The purpose of this figure to show that the three arms of CD163 form very different interactions with Hb and HpHb and our view is that the current representation delivers this most effectively. The few specialist readers who require the level of detail suggested by the reviewer will be free to download the coordinates to have a look for themselves. For most readers, the figures which we include in the manuscript will be far more informative.

Minor Revisions

• In Lines 131-133, the authors describe the aligned SRCR5-9 base architectures as having "an extremely similar base architecture, with an overall RMSD of 1.9 Å." An overall RMSD of 1.9 Å represents a moderate structural deviation, so the use of the word "extremely" should be tempered or removed. Additionally, the authors should elaborate on the specific base differences between the CD163-HpHb and CD163-Hb structures in the Figure 2B legend to better define what "little base movement" entails in this context.

We have shown the overlay of the base in Figure 2B so that readers can see for themselves how similar the trimeric base is in these two complexes.

We have removed the word 'extremely' as we agree that this is subjective word and best left to the reader to decide if they are similar.

We have noted in lines 137-139 that these adjustments, which are confident that the reader will see in Figure 2B are small, can be achieved by rocking of the subunits relative to one another in the way previously observed.

• In Lines 181-185, the authors describe a complex mechanism of tilting and flexibility around the calcium-mediated interface joining SRCR5-7 and the interface between SRCR2 and 3. These movements are difficult to visualize from the text alone. The authors should consider providing a schematic illustration or diagram to clarify these dynamic transitions.

We are confident that the rocking between SRCR2 and 3, which is a new conclusion in this manuscript, is very clear from Figure 2C.

We agree that the rocking around the calcium-mediated interface is not shown. However, this is not a new conclusion from this manuscript but is seen in our previous HpHb-bound CD163 structure (Zhou et al 2025). We have clarified this in line 190.

• The authors utilized different molar ratios of CD163 to Hb for the analytical ultracentrifugation experiments versus the cryo-EM sample preparation. Do the authors have explanations for these differing ratios?

As the reviewer will see in Figure S4, we analysed complexes with 7μM CD163 and 40μM of either Hb or HpSPHb. In the case of the Hb complex, this generated a sharp peak for monodispersed trimeric complex and one Hb tetramer. In the case of HpSPHb, a distribution of liganded states is seen, which made the data harder to interpret. We therefore repeated at a lower concentration of HpSPHb of 3μM to increase the fraction of liganded CD163 and more readily observe the different states. In the case of Hb, 3μM was insufficient to

generate liganded CD163 due to the lower affinity. We have presented a set of comparable data.

• In Line 219 of the Methods, the authors state they mixed CD163 trimer with "Hb dimer" at a 1:1 molar ratio. Did the authors really use Hb dimer to make CD163-Hb(tetramer) complex for cryo-EM analysis? Or is it a typo?

We agree with the reviewer that this was confusing. We did not use Hb dimer as this cannot be readily stabilised. We used a 2:1 molecular ratio of CD163 trimer to Hb tetramer, aiming to give an excess of receptor rather than the smaller ligand. We have corrected this in line 229.

Reviewer #3: The study by Zhou & Higgns reports the first structures of the CD163 in complex with hemoglobin. The authors use CryoEM in combination with binding and uptake assays to characterize the differences in CD163's affinity and engagement with substrates HpSBHb and Hb.

The study is straightforward, and the experiments appear well carried out. There are some modest points where the analysis can be more thorough, but I expect these to be easily addressed in a revision.

We thank the reviewer for their positive review of the manuscript.

I am a bit confused about the stoichiometry of the CD163 base. Dimers of CD163 are observed in CryoEM samples prepared without substrate or with HpSPHb. No dimer is noted here. This should be discussed, as it has significant implications for the assembly of the complexes.

Relatedly, in the methods (line 271) it is stated that Hb binds trimeric CD163. Is this a conclusion solely based on the structure?

The reviewer is correct in noting that dimers and trimers of CD163 are observed in samples lacking ligand or in the presence of HpSPHb. However, we only observed trimeric forms bound to Hb. Our cryoEM data (i.e. Figure S2, which shows the three-dimensional classes observed) is supported by our AUC data (Figure 2A and Figure S4), both of which confirm that trimeric CD163 is required to bind to the lower affinity Hb. This, together with its implications, are presented and discussed in lines 114-130. We have also added a new sentence to the discussion in lines 193-196. Our conclusion is that lower affinity ligands require a trimeric arrangement to bind sufficiently tightly to allow uptake.

It is curious that the Hb chains appear to interaction with different arms between the HpSPHb and Hb complexes. Are the Hb-SRCR domain interactions similar or different between complexes? Alternatively, are there obvious differences between the two complexes to explain this difference in engagement?

It is indeed very interesting that very different interactions are mediated between the CD163 arms and the Hb component of the Hb tetramer and the HpSPHb complex. We outline these differences in lines 147-162 and show them in Figure 3AB. Here we show that Hb and HpSPHb sit in different positions with different orientations relative to the CD163 base. We discuss in the manuscript how this protein can use multiple small binding sites with low affinity, combining them to generate a higher affinity binding pocket (lines 182-186), which is one of the amazing features of this molecule.

In Figure 1B, there appears to be significant uptake of Hb in the absence of CD163. Was this background uptake corrected for in the EC50 calculation?

We confirm that background binding was subtracted before calculation of EC50 values.

In the introduction, HpSPHb is defined as describing a structural region within the larger complex between Hp and a Hb dimer. However, in the remainder of the paper this same term is used to describe a construct with a truncated Hp that has been engineered, expressed, and assembled to minimally capture this structural region. This should be clarified.

We have added a definition of HpSPHb in lines 88-9

The authors mention free Hb is normally low, but elevated in several disease states. If known, it would be very helpful to give molar concentrations of Hb in these states for comparison to measured CD163 affinities.

We have added a section into the introduction in lines 70-72 to give a ballpark estimate. These concentrations are higher than the K_D for CD163, indicating that uptake will occur.

Relatedly, in the introduction, circulating Hp is given in mg/ml. However, it would be helpful to give this in molar.

We agree with the reviewer that using molar measurements would be better. The challenge is that many of the studies use mg/ml and are not clear which isoforms of Hp they are studying as these have different sizes and different multimeric states. We have estimated the molarity assuming 100kDa for an isoform 1 dimer and added this in line 69 as an indicator of approximate molarity.
